# Effects of *Tamarindus indica* Fruit Pulp Extract on Abundance of HepG2 Cell Lysate Proteins and Their Possible Consequential Impact on Metabolism and Inflammation

**DOI:** 10.1155/2013/459017

**Published:** 2013-12-25

**Authors:** Ursula R. W. Chong, Puteri S. Abdul-Rahman, Azlina Abdul-Aziz, Onn H. Hashim, Sarni Mat-Junit

**Affiliations:** ^1^Department of Molecular Medicine, Faculty of Medicine, University of Malaya, 50603 Kuala Lumpur, Malaysia; ^2^University of Malaya Centre for Proteomics Research, Medical Biotechnology Laboratory, Faculty of Medicine, University of Malaya, 50603 Kuala Lumpur, Malaysia

## Abstract

The fruit pulp extract of *Tamarindus indica* has been reported for its antioxidant and hypolipidemic properties. In this study, the methanol extract of *T. indica* fruit pulp was investigated for its effects on the abundance of HepG2 cell lysate proteins. Cell lysate was extracted from HepG2 cells grown in the absence and presence of the methanol extract of *T. indica* fruit pulp. Approximately 2500 spots were resolved using two-dimensional gel electrophoresis and the abundance of 20 cellular proteins was found to be significantly reduced. Among the proteins of reduced abundance, fourteen, including six proteins involved in metabolism (including ethanolamine phosphate cytidylyltransferase), four mitochondrial proteins (including prohibitin and respiratory chain proteins), and four proteins involved in translation and splicing, were positively identified by mass spectrometry and database search. The identified HepG2 altered abundance proteins, when taken together and analyzed by Ingenuity Pathways Analysis (IPA) software, are suggestive of the effects of *T. indica* fruit pulp extract on metabolism and inflammation, which are modulated by LXR/RXR. In conclusion, the methanol fruit pulp extract of *T. indica* was shown to cause reduced abundance of HepG2 mitochondrial, metabolic, and regulatory proteins involved in oxidative phosphorylation, protein synthesis, and cellular metabolism.

## 1. Introduction

*Tamarindus indica* or tamarind is a tropical fruit tree native to the African savannahs but it can now be found in many tropical countries. It is categorized as a monospecific genus in the family of Leguminosae. The sweet and sour taste of its fruit pulp is used to add flavor to local cuisines. Besides culinary, tamarind is also used in traditional medicine as laxative, diuretic, antibacterial agents as well as in treatment of fever and malarial infections [1, 2]. Previous biochemical analyses have demonstrated that extracts of *T. indica* possess high antioxidant activities [3, 4]. In addition, *T. indica* extracts have also been shown to reduce the levels of blood cholesterol and triacylglycerol in hypercholesterolemic hamsters [3] and in humans [5]. However, the molecular mechanisms of the anti-inflammatory and hypolipidemic effects of the fruit remain elusive.

In our previous study, the methanol extract of *T. indica* fruit pulp was shown to alter the expression of more than a thousand genes in HepG2 cells, many of which are associated with lipid metabolism [6]. Our recent study also showed that the fruit pulp extract was able to alter the secretion of alpha enolase, apolipoprotein A-1, transthyretin, and rab GDP dissociation inhibitor beta from HepG2 cells, which may account for the lipid-lowering effects of the fruit. These effects were hypothesized to occur via activation of LXR/RXR [7].

The lipid-lowering properties of *T. indica* fruit pulp are likely attributed to the presence of polyphenols in its extract. The methanol extract of *T. indica* fruit pulp contains predominantly proanthocyanidins of various forms, including (+)-catechin [4, 8] and (−)-epicatechin, along with taxifolin, apigenin, eriodictyol, luteolin, and naringenin [4]. Proanthocyanidins from grape seeds have been shown to lower the levels of blood cholesterol and triacylglycerol in hamsters through excretion of bile [9]. In addition, other polyphenols like naringenin [10] and tea catechins [11] have also been shown to have effects on transcriptional regulation of hepatic lipid metabolism in rats and humans.

In this study, the methanol extract of *T. indica* fruit pulp was further investigated for its effects on the abundance of HepG2 cell lysate proteins by two-dimensional gel electrophoresis and densitometry. Identification of HepG2 cell lysate proteins that are significantly altered in abundance may help to improve our understanding of the metabolic pathways and molecular mechanisms that are affected by the fruit pulp.

## 2. Materials and Methods

### 2.1. Preparation of Methanol Extract of the *T. indica* Fruit Pulp

*T. indica* fruit pulps were collected at Universiti Putra Malaysia and the fruits were identified through comparison with a registered voucher specimen (KLU 45976) deposited in the Herbarium of Institute of Biological Sciences, University of Malaya. *T. indica* fruit pulp extract was prepared as previously described [6], with slight modifications. Briefly, ripe fruit pulp of *T. indica* was separated from the seeds, air-dried and powdered. The powdered fruit pulp (10 g) was placed in a conical flask and soaked in 200 mL methanol at room temperature. The mixture was then stirred with a magnetic stirrer for 1 h and kept in the dark for 24 h. The resulting extract was then filtered and dried in a rotary evaporator and finally redissolved in 10% DMSO. Samples were kept at −20°C until further analysis.

### 2.2. Cell Culture

Human hepatoma HepG2 cell line (ATCC, Manassas, VA, USA) was grown in a complete medium consisting of Dulbecco's modified Eagle's medium (DMEM) supplemented with 5 mM glucose, 10% foetal bovine serum (FBS; HyClone, Australia), 0.37% (w/v) sodium bicarbonate and 0.48% (w/v) HEPES, pH 7.4, in a CO_2_ humid incubation chamber at 37°C.

### 2.3. Treatment of HepG2 Cells with *T. indica* Fruit Pulp Extract and Preparation of Cell Lysate

HepG2 cells were seeded at a density of 9.0 × 10^6^ in a 75 cm^2^ flask for 18–24 h, followed by extensive washing with phosphate-buffered saline (PBS). The cells were then incubated for 24 h in serum-free medium in the presence of 0.02% DMSO (vehicle) as controls or a final concentration of 60 *μ*g/mL methanol extract of *T. indica* fruit pulp. The cells were then lysed in rehydration buffer (7 M urea, 2 M thiourea, 2% w/v CHAPS, 0.5% v/v IPG buffer, orange G, protease inhibitor) and protein concentration of lysate was determined using the Bradford assay kit (Bio-rad, Hercules, CA, USA).

### 2.4. Two-Dimensional Gel Electrophoresis (2D-GE)

Forty *μ*g of cell lysate proteins were cleaned using the 2D clean-up kit (GE, Piscataway, USA). The resulting protein pellet was then reconstituted in rehydration solution containing 7 M urea, 2 M thiourea, 2% w/v CHAPS, 0.5% v/v IPG buffer, orange G, and protease inhibitor. Immobiline pH gradient strip (13 cm, non-linear, pH 3–10, GE Healthcare, Uppsala, Sweden) was then rehydrated in the reconstituted protein sample for 18 h at room temperature. First dimension separation was performed on an Ettan IPGphor III (GE, Uppsala, Sweden) ran on the following conditions: (i) 500 V, 1 hr 10 mins, step and hold; (ii) 1000 V, 1 hr, gradient; (iii) 8000 V, 2 hrs 30 mins, gradient and (iv) 8000 V, 55 mins, step and hold. The strips were then equilibrated in SDS equilibration buffer containing 6 M urea, 75 mM Tris-HCl, pH 8.8, 29.3% v/v glycerol, 2% w/v SDS, 0.002% w/v bromophenol blue, and 1% w/v dithiothreitol (DTT) for 15 min, followed by a second equilibration using the same buffer containing 4.5% w/v iodoacetamide instead of DTT for another 15 min. Second dimension separation was carried out on 12.0% polyacrylamide gel using the SE 600 Ruby electrophoresis system (GE Healthcare, Uppsala, Sweden) at a constant voltage of 50 V and 40 mA/gel for 20 min, and then switched to 500 V and 40 mA/gel until the bromophenol blue marker was 1 mm away from the bottom of the gel. Gels were silver-stained with PlusOne Silver Staining Kit (GE Healthcare, Uppsala, Sweden) and scanned using the ImageScanner III (GE Healthcare, Uppsala, Sweden).

### 2.5. Image and Data Analyses

Gel images were analyzed using the ImageMaster 2D Platinum V 7.0 software (GE Healthcare, Uppsala, Sweden). Briefly, the 2D gel images were subjected to spot detection and quantification in the differential in-gel analyses module. Protein spots were normalized using percentage of volume to minimize variations between gels within the same group. Statistically significance (*P* < 0.05, Student's *t*-test) and presence in all 6 gels were the two criteria for acceptance of protein spots of altered abundance. Selected spots were filtered based on an average expression level change of at least 1.5-fold.

### 2.6. In-Gel Tryptic Digestion

Protein spots were excised manually from 2D-GE gels and washed with 100 mM NH_4_HCO_3_ for 15 min. The gel plugs were then destained twice with 15 mM potassium ferricyanide/50 mM sodium thiosulphate with shaking. They were then reduced with 10 mM DTT at 60°C for 30 min and alkylated with 55 mM iodoacetamide in the dark at room temperature for 20 min. The plugs were later washed thrice with 500 *μ*L of 50% ACN/50 mM NH_4_HCO_3_ for 20 min, dehydrated with 100% ACN for 15 min, and dried using the SpeedVac. The gel plugs were finally digested in 6 ng/*μ*L trypsin (Pierce, Rockford, IL USA) in 50 mM NH_4_HCO_3_ at 37°C for at least 16 h. Peptide mixtures were extracted twice with 50% ACN and 100% ACN, respectively, and finally concentrated using the Speedvac until completely dry. Dried peptides were then kept at −20°C or reconstituted with 10 *μ*L of 0.1% TFA, prior to desalting using the Zip Tip C18 micropipette tips (Millipore, Billerica, MA, USA).

### 2.7. Mass Spectrometry and Database Search

Trypsin digested peptide mixture was analyzed using an Applied Biosystems 4800 Plus MALDI-TOF/TOF (Foster City, CA, USA). The peptide mixture was crystallized with alpha-cyano-4-hydroxycinnamic acid matrix solution (10 mg/mL, 70% ACN in 0.1% (v/v) TFA aqueous solution) and spotted onto a MALDI target (192-well) plate. The MS results were automatically acquired with a trypsin autodigest exclusion list and 20 most intense ions were selected for MS/MS analysis. Data analysis was carried out using the GPS Explorer software (Applied Biosystems, CA, USA) and database search using the in-house MASCOT program (Matrix Science, London, UK). Combined MS and MS/MS searches were conducted with the following settings: Swiss-Prot database, *Homo sapiens*, peptide tolerance at 200 ppm, MS/MS tolerance at 0.4 Da, carbamidomethylation of cysteine (variable modification) and methionine oxidation (variable modifications). A protein is considered identified when a MASCOT score of higher than 55 and *P* < 0.05 were obtained from the MS analysis.

### 2.8. Functional Analyses Using Ingenuity Pathways Analysis (IPA) Software

Functional analyses to predict networks that are affected by the differentially expressed proteins were carried out using Ingenuity Pathways Analysis (IPA) software (Ingenuity Systems, http://www.ingenuity.com/). Details of the proteins, their quantitative expression values (fold change difference of at least 1.5) and *P*  values (*P* < 0.05) were imported into the IPA software. Each protein identifier was mapped to its corresponding protein object and was overlaid onto a global molecular network developed from information contained in the Ingenuity Knowledge Base. Network predictions based on the protein input were generated algorithmically by utilizing the information contained in the Ingenuity Knowledge Base. Right-tailed Fischer's exact test was used to calculate a *P*  value indicating the probability that each biological function assigned to the network is due to chance alone.

### 2.9. Validation of Proteins of Reduced Abundance

Three of the altered proteins, ethanolamine phosphate cytidylyltransferase (PCYT2) and NADH dehydrogenase (ubiquinone) 1 alpha subcomplex subunit 10 (NDUFA10) and ubiquinol-cytochrome-c reductase complex core protein 2 (UQCRC2) were selected for validation using Western blotting. HepG2 cells (3 × 10^6^) were treated with 60 *μ*g/mL *T. indica* fruit pulp extract for 24 h. The cells were then trypsinized and lysed with RIPA buffer (150 mM NaCl; 50 mM Tris-HCl, pH 7.4; 1 mM EDTA; 1% Triton X-100; 1% sodium deoxycholate; 0.1% SDS). Total cell lysate proteins were quantified using BCA assay kit (Pierce, Rockford, IL, USA). Forty micrograms of cell lysate protein was separated on a 12.5% SDS-PAGE and transferred onto a PVDF membrane with 0.45 *μ*m pore size (Thermo Scientific, IL, USA) at 100 V, 110 mA for 90 minutes. The blot was then blocked overnight and developed against anti-PCYT2 (ab126142, rabbit polyclonal, Abcam, UK), anti-NDUFA10 (ab103026, rabbit polyclonal, Abcam, UK), anti-UQCRC2 (ab103616, rabbit polyclonal, Abcam, UK), and anti-beta actin (ab8227, rabbit polyclonal, Abcam, UK) as the loading control using the WesternDot 625 Goat Anti-Rabbit Western Blot kit (Invitrogen, Oregon, USA). Quantification of the band intensity was calculated using ImageJ software.

## 3. Results

### 3.1. 2D-GE Analysis of HepG2 Cell Lysate Proteins

**Figure 1:**
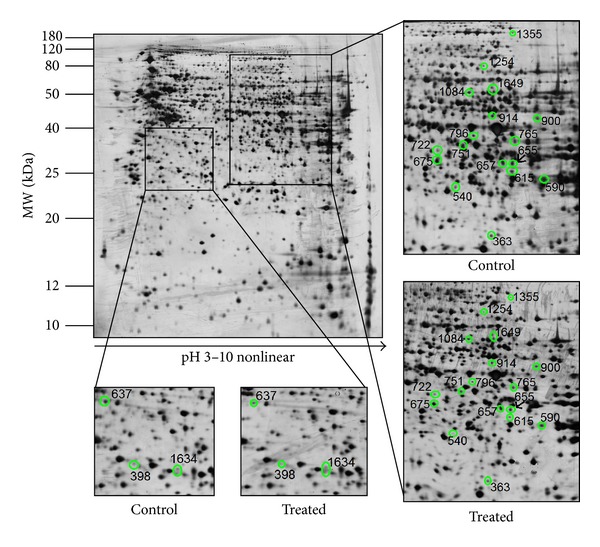
A representative proteome map of HepG2 cell lysate. Approximately 2500 spots per gel within the pH 3–10 range for cell lysate of HepG2 cells were detected. Twenty spots were altered in abundance (circled and labelled) and they were all significantly reduced in abundance (*P* < 0.05).

Analysis of HepG2 cell lysate by 2D-GE resolved more than 2500 protein spots in each gel.  Figure 1 demonstrates typical 2D-GE resolved patterns of the cell lysate proteins from controls and HepG2 cells treated with the fruit pulp extract of *T. indica*. The percentage of volume contribution of each spot was then determined using ImageMaster 2D Platinum V 7.0 software, and the fold change, if any, was acquired. Protein spots that were present in all gels (*n* = 6) and showed significant differences in their abundance (*P* < 0.05) in treated and nontreated cells by more than 1.5-fold were selected. Based on these criteria, the altered abundance of 20 protein spots were detected when HepG2 cells were exposed to the fruit pulp extract of *T. indica* (Table 1).

### 3.2. Identification of Altered Abundance HepG2 Cell Lysate Proteins

Among the 20 protein spots that were significantly reduced in abundance, 14 were successfully identified by mass spectrometry and database search (Table 2). Six spots (protein spot ID 398, 615, 637, 1254, 1355, and 1649) were considered not successfully identified as their scores were lower than the cut-off value for positive inclusion criteria. The 14 identified proteins may be grouped according to their biological processes using the UniProt Protein Knowledgebase (UniProtKB). NADH dehydrogenase (ubiquinone) 1 alpha subcomplex subunit 10, ubiquinol-cytochrome-c reductase complex core protein 2, and NADH dehydrogenase (ubiquinone) flavoprotein 1 were grouped under “mitochondrial respiratory chain” category. Three proteins, that is, eukaryotic translation initiation factor 3 subunit 3, elongation factor Tu, and tyrosyl-tRNA synthetase, are involved in “protein synthesis.” Another six proteins were categorized under “metabolism,” with glyceraldehyde-3-phosphate dehydrogenase and GDP-L-fucose synthetase being involved in carbohydrate metabolism, GMP reductase 2 and UMP synthase in nucleotide and nucleoside metabolism, S-methyl-5-thioadenosine phosphorylase in polyamine metabolic process, and ethanolamine phosphate cytidylyltransferase in biosynthesis of phospholipids. Prohibitin, on the other hand, was categorized under “cell proliferation and differentiation,” while heterogeneous nuclear ribonucleoprotein H3 does not belong to any of the above groups and therefore categorized as “others.”

### 3.3. Biological Processes and Pathway Interaction Analysis

**Figure 2:**
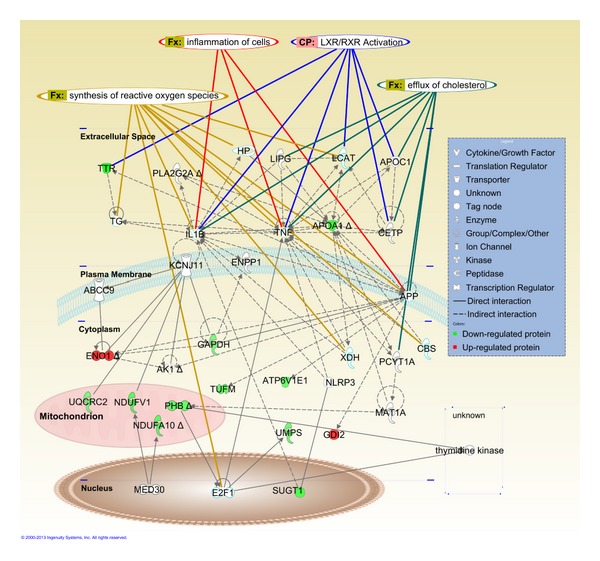
IPA graphical representation of the molecular relationships between HepG2 secreted and cytosolic proteins after treatment. The network is displayed graphically as nodes (proteins) and edges (the biological relationships between the nodes). Nodes in red indicate up-regulated proteins while those in green represent down-regulated proteins. Nodes without colors indicate unaltered expression. Various shapes of the nodes represent functional class of the proteins. Edges are displayed with various labels that describe the nature of the relationship between the nodes. Transthyretin, TTR; thyroglobulin, TG; interleukin-1 beta, IL1B; tumour necrosis factor, TNF; apolipoprotein A-1, APOA1; apolipoprotein C-1, APOC1; lecithin cholesterol acyltransferase, LCAT; endothelial lipase, LIPG; haptoglobin, HP; phospholipase A2, PLA2G2A; cholesterylester transfer protein, CETP; ATP-binding cassette transporter sub-family C member 9, ABCC9; ATP-sensitive inward rectifier potassium channel 11, KCNJ11; ectonucleotide pyrophosphatase/phosphodiesterase family member 1, ENPP1; amyloid precursor protein, APP; glyceraldehyde-3-phosphate dehydrogenase, GAPDH; alpha enolase, ENO1; adenylate kinase, AK1; ubiquinol-cytochrome-c reductase complex core protein 2, UQCRC2; xanthine dehydrogenase, XDH; cystathionine beta-synthase, CBS; methionine adenosyltransferase I, alpha, MAT1A; rab GDP dissociation inhibitor beta, GDI2; NLR (nucleotide-binding domain and leucine rich repeat containing family) family, pyrin domain containing 3, NLRP3; Vacuolar ATP synthase subunit E, ATP6V1E1; elongation factor Tu, TUFM; prohibitin, PHB; choline-phosphate cytidylyltransferase A, PCYT1A; uridine 5′-monophosphate synthase, UMPS; NADH dehydrogenase (ubiquinone) 1 alpha subcomplex subunit 10, NDUFA10; NADH dehydrogenase (ubiquinone) flavoprotein 1, NDUFV1; mediator of RNA polymerase III transcription subunit 30, MED30; transcription factor E2F1, E2F1; suppressor of G2 allele of SKP1 homolog, SUGT1.

The cell lysate proteins of altered abundance, when analyzed using Ingenuity Pathways Analysis (IPA) software, generated a single network on “Hereditary disorder, metabolic disease, molecular transport,” with a score of 48 (Table 3). Mitochondrial dysfunction was ranked with the highest significance (*P* < 3.65 × 10^−4^) from a canonical pathway analysis. When the data was reanalyzed to include our earlier reported results of HepG2 proteins that were differentially secreted when the cells were exposed to the *T. indica* fruit pulp extract [7], the software identified “Lipid Metabolism, Molecular Transport and Small Molecule Biochemistry” as top putative network (score of 31) that links the proteins of altered abundance with other interactomes, and gluconeogenesis I became the top canonical pathway (*P* < 4.67 × 10^−4^) (Table 3).  Figure 2 shows a graphical representation of the predicted molecular relationships between HepG2 proteins that were altered in abundance in response to exposure to *T. indica* fruit pulp extract. Other than the lipid-related interactomes, the HepG2 proteins of altered abundance were shown to be associated with inflammation-related molecules like tumour necrosis factor (TNF) and interleukin-1 beta (IL-1*β*). Two transcription regulators, mediator of RNA polymerase III transcription subunit 30 (MED30), and transcription factor E2F1 (E2F1) were also shown to be interconnected with the proteins.

### 3.4. Confirmation of the Effects of *T. indica* Fruit Pulp Extract on HepG2 Proteins

**Figure 3:**
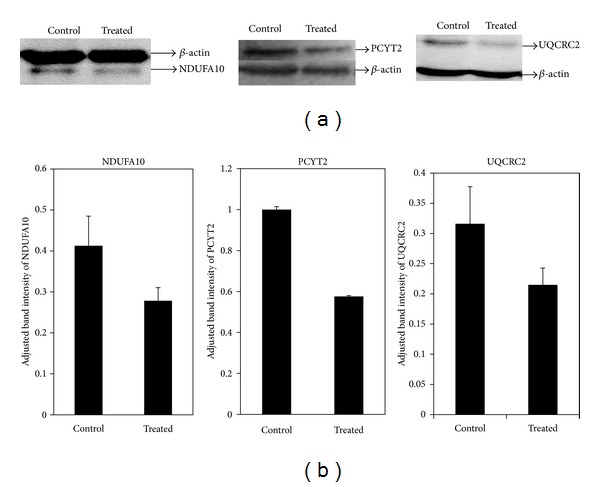
Western blot analyses of NDUFA10, PCYT2, and UQCRC2 of HepG2 cells. (a) Western blot cropped images of NDUFA10, PCYT2, UQCRC2, and beta actin bands detected by antisera against the respective proteins; (b) densitometry analyses of Western blot using ImageJ software. Assay was done in triplicate and data are represented as mean ± standard deviation.

To validate the effects of the *T. indica* fruit pulp extract on HepG2 proteins, Western blotting was performed using antisera raised against the cellular proteins. In view of the scarce amount of HepG2 cell lysate protein extract that was generated in this study, three proteins, that is, ethanolamine phosphate cytidylyltransferase (PCYT2), NADH dehydrogenase (ubiquinone) 1 alpha subcomplex subunit 10 (NDUFA10), and ubiquinol-cytochrome-c reductase complex core protein 2 (UQCRC2) that represent those involved in the mitochondrial and metabolic activities were selected. Densitometry scanning of bands detected by the antisera showed that the abundance of PCYT2, NDUFA10, and UQCRC2 was indeed lower in HepG2 cells treated with the *T. indica* fruit pulp extract compared to the controls, with fold differences of –1.7, –1.5, and –1.5, respectively (Figure 3).

## 4. Discussion

In this study, the abundance of 20 cell lysate proteins was found to be significantly reduced when HepG2 cells were exposed to the *T. indica* fruit pulp extract. Fourteen of the proteins were identified by mass spectrometry and database search, and the reduced abundance of three of the HepG2 proteins was subsequently validated by Western blotting. Due to limited availability of antibodies and HepG2 cell lysate protein extract, validation was performed on the three selective proteins that are representative of separate mitochondrial functions and metabolic activities.

Among the identified HepG2 proteins, three components of the mitochondrial respiratory chain, namely, ubiquinol-cytochrome-c reductase complex core protein 2 (UQCRC2), NADH dehydrogenase (ubiquinone) 1 alpha subcomplex subunit 10 (NDUFA10), and NADH dehydrogenase (ubiquinone) flavoprotein 1 (NDUFV1), were found to be reduced in abundance when HepG2 cells were exposed to *T. indica* fruit pulp extract. UQCRC2 belongs to complex III of the mitochondrial respiratory chain while NDUFA10 and NDUFV1 are components of complex I. In mitochondria, only complex I [12, 13] and complex III [14] of the respiratory chain are known to produce reactive oxygen species (ROS). Hence, the decreased amount of the three mitochondrial components may indicate reduced production of free radicals, although the functionality of the mitochondria is yet to be confirmed. Nevertheless, earlier studies in obese rat muscles have shown that reduced levels of respiratory chain complex I and diminished ROS production that were induced by chronic supplementation with grape seed proanthocyanidins did not affect the function of the mitochondria [15].

Prohibitin is another mitochondrial protein that was shown to be of reduced abundance in HepG2 cells exposed to *T. indica* fruit pulp extract. The protein that was initially thought to be a negative regulator of cell proliferation has been demonstrated to play a role in biogenesis and function of mitochondria [16]. In a study of the nematode *Caenorhabditis elegans*, prohibitin complex was shown to promote longevity by modulating mitochondrial function and fat metabolism [17]. Deficiency of prohibitin prolongs the lifespan of *C. elegans* with compromised mitochondrial function or fat metabolism. In this study, depletion of prohibitin in HepG2 cells upon exposure to the fruit pulp extract of *T. indica* appears to suggest a similar mechanism in an attempt to extend lifespan of the cells.

In addition to the mitochondrial proteins, the glycolytic enzyme glyceraldehyde 3-phosphate dehydrogenase (GAPDH), as well as GDP-L-fucose synthetase (TSTA3), also appeared to be down-regulated after treatment with *T. indica* fruit pulp extract. Oxidative stress is known to induce up-regulation of GAPDH levels [18, 19]. Hence, the reduced amount of GAPDH in this case may possibly indicate a state of repressed oxidative stress. This, together with the earlier data on the reduced mitochondrial respiratory chain proteins, may shed some light on molecular mechanisms involved in the well acclaimed antioxidant properties of *T. indica* [3, 4].

The fruit pulp extract of *T. indica* also appeared to cause decreased abundance of proteins involved in the metabolism of nucleic acids and polyamines in HepG2 cells. To the best of our knowledge, these have not been previously reported and their rationale is not quite understood. On the other hand, decreased abundance of ethanolamine phosphate cytidylyltransferase (PCYT2), the rate-limiting enzyme which catalyzes conversion of phosphoethanolamine to cytidylylphosphoethanolamine in the biosynthesis of phosphatidylethanolamine, in HepG2 cells exposed to the fruit extract of *T. indica* may compromise availability of the phospholipid, which stores arachidonic acid for the production of prostaglandins. This could possibly explain the anti-inflammatory action of *T. indica* that was earlier reported [20].

In addition, eukaryotic translation initiation factor 3 subunit 3 (eIF3H), tyrosyl-tRNA synthetase (YARS), elongation factor Tu (EFTU), and heterogenous nuclear ribonucleoprotein H3 (hnRNP H3), which are generally involved in protein synthesis and splicing, also appeared to be down-regulated in HepG2 cells treated with the *T. indica* extract. These proteins may be key regulatory points of action of the fruit pulp extract of *T. indica.* Suppression of these proteins by the extract may reflect the underlying epigenetic mechanism that ultimately caused the reduced expression of all the mitochondrial and metabolic proteins and enzymes.

Subjecting the altered abundance proteins to IPA analysis generated a single network on “Hereditary disorder, metabolic disease, molecular transport,” which ranked mitochondrial dysfunction with the highest significance (*P* < 3.65 × 10^−4^). However, “Lipid Metabolism, Molecular Transport, Small Molecule Biochemistry” became the top network involved when IPA was reanalyzed to include proteins that were previously shown to be differentially secreted by HepG2 cells treated with the same fruit extract [7]. This network was not generated in the earlier analysis as PCYT2 was the sole cellular protein involved in lipid metabolism that was affected when HepG2 cells were exposed to *T. indica* fruit pulp extract. In our earlier IPA analysis of secreted proteins of altered abundance from HepG2 exposed to the *T. indica* fruit pulp extract, a score of 9 was obtained [7], and this improved to 31 when the data were reanalyzed to include cell lysate proteins of reduced abundance, signifying markedly higher probability. In addition, the IPA software also identified tumour necrosis factor (TNF) and interleukin-1 beta (IL-1*β*), both of which are potent inflammatory mediators, as interactomes in the network affected by *T. indica* (Figure 2). This further supports our earlier speculation on the molecular mechanism involved in anti-inflammatory effects of *T. indica*.

The lipid-lowering effects induced by plant polyphenols have been reported by many. In fact, there is an upcoming trend of research revealing the potentials of plant polyphenols in regulating *in vitro* and *in vivo* metabolic processes. The methanolic extract of *T. indica* fruit pulp contains proanthocyanidins, which constitutes more than 73% of its total phenolic content [4]. Proanthocyanidin modulates activation of LXRs [9], which are oxysterol-activated nuclear receptors that control cholesterol homeostasis by modifying expression of genes involved in cholesterol absorption and efflux from peripheral tissues. This process is mediated through ABCA-1-mediated cholesterol efflux and ABCG5/8-mediated cholesterol excretion and absorption. Interestingly, LXRs also regulate genes essentially involved in inflammation (Figure 2). As speculated earlier, the anti-inflammatory action of *T. indica* fruit pulp may possibly occur by inhibition of synthesis of phospholipids and hence depleting the arachidonic acid reservoir for the generation of prostanoid inflammatory mediators.

## 5. Conclusion 

The methanol fruit pulp extract of *T. indica* was shown to cause reduced abundance of HepG2 mitochondrial, metabolic, and regulatory proteins involved in oxidative phosphorylation, protein synthesis, and cellular metabolism. The cellular proteins, when taken together with the earlier HepG2 secreted proteins of altered abundance, are suggestive of the effects of the fruit pulp extract of *T. indica* on inflammation and lipid metabolism, which are modulated by LXRs.

## Figures and Tables

**Table 1:** Average percentage of volume of spots.

Spot ID	Average percentage of volume ± SEM	
	Control	Treated
363	0.0273 ± 0.0016	0.0187 ± 0.0019
398	0.0214 ± 0.0021	0.0134 ± 0.0020
540	0.0128 ± 0.0019	0.0073 ± 0.0011
590	0.0892 ± 0.0073	0.0609 ± 0.0039
615	0.0240 ± 0.0023	0.0157 ± 0.0016
637	0.0254 ± 0.0033	0.0156 ± 0.0011
655	0.0499 ± 0.0033	0.0328 ± 0.0056
657	0.0655 ± 0.0088	0.0360 ± 0.0035
675	0.0660 ± 0.0067	0.0440 ± 0.0017
722	0.0223 ± 0.0022	0.0144 ± 0.0014
751	0.0420 ± 0.0038	0.0235 ± 0.0027
765	0.0470 ± 0.0031	0.0315 ± 0.0040
796	0.0162 ± 0.0011	0.0093 ± 0.0009
900	0.0394 ± 0.0048	0.0206 ± 0.0044
914	0.0279 ± 0.0015	0.0177 ± 0.0012
1084	0.0305 ± 0.0015	0.0209 ± 0.0017
1254	0.0154 ± 0.0018	0.0078 ± 0.0007
1355	0.0107 ± 0.0018	0.0054 ± 0.0010
1634	0.0746 ± 0.0125	0.0435 ± 0.0031
1649	0.0639 ± 0.0069	0.0416 ± 0.0032

The protein spots show significant differences in their abundance (*P* < 0.05) in *T. indica*-treated and nontreated (control) HepG2 cells by more than 1.5-fold.

**Table 2:** List of cell lysate proteins of altered abundance in *T. indica* fruit extract-treated cells identified by MALDI-TOF/TOF MS/MS.

Spot no.	Protein description	Acc. no.	Score	pI/Mw (kDa)	Av. % FC^a^	% Cov^b^	Matched peptide sequences	Functional category
657	NADH dehydrogenase (ubiquinone) 1 alpha subcomplex subunit 10 (CI-42 kD)	O95299	194	8.67/40.73	−1.8	44	117–122; 13–139; 140–161; 253–261; 290–295	Mitochondrial respiratory chain
765	Ubiquinol-cytochrome-c reductase complex core protein 2, mitochondrial precursor core protein II	P22695	537	8.74/48.41	−1.5	28	71–84; 163–183; 184–196; 200–217; 232–241	
900	NADH dehydrogenase (ubiquinone) flavoprotein 1, mitochondrial precursor (CI-51 kD)	P49821	275	8.51/50.79	−1.9	33	72–81; 153–159; 160–174; 376–386; 441–449	
796	Ethanolamine-phosphate cytidylyltransferase	Q99447	339	6.44/43.81	−1.7	38	185–199; 262–271; 333–348	Phospholipid biosynthesis
590	Glyceraldehyde-3-phosphate dehydrogenase (GAPDH)	P04406	192	8.57/36.03	−1.5	18	235–248; 310–323	Carbohydrate metabolism
722	GDP-L-fucose synthetase	Q13630	280	6.13/35.87	−1.5	49	26–44; 82–88; 90–107; 200–214; 291–297; 307–320	
655	GMP reductase 2	Q9P2T1	386	6.79/37.85	−1.5	52	70–78; 178–189; 192–213; 277–286; 278–286; 292–298; 292–306	Nucleotide and nucleoside metabolism
914	Uridine 5′-monophosphate synthase (UMP synthase)	P11172	379	6.81/52.20	−1.6	37	6–22; 30–41; 146–155; 353–363; 389–405; 460–467; 469–477	
363	S-methyl-5-thioadenosine phosphorylase (MTAP)	Q13126	253	6.75/31.23	−1.5	43	12–29; 72–82; 83–99; 100–116; 134–147; 181–187; 272–282	Polyamine metabolism
1634	Prohibitin	P35232	66	2.27/29.79	−1.7	21	134–143	Cell proliferation and differentiation
675	Eukaryotic translation initiation factor 3 subunit 3 (elF3H)	O15372	279	6.09/39.91	−1.5	43	52–75; 242–249; 260–265; 304–313; 314–331	Protein biosynthesis
751	Elongation factor Tu, mitochondrial precursor (EF-Tu)	P49411	114	7.26/49.51	−1.8	29	105–120; 239–252	
1084	Tyrosyl-tRNA synthetase, cytoplasmic (TyrRS)	P54577	96	6.61/59.11	−1.5	21	85–93; 179–189; 433–450	
540	Heterogenous nuclear ribonucleoprotein H3 (hnRNP H3)	P31942	409	6.37/36.90	−1.7	39	56–67; 85–90; 98–104; 206–222; 223–232; 262–287; 288–301	Others

^a^Negative value signifies down-regulation in terms of fold-differences. All ratios are statistically significant with *P* < 0.05 (Student's *t*-test).

^b^% Coverage of the identified sequence.

**Table 3:** Top network and canonical pathways generated by Ingenuity Pathways Analysis.

Proteins subject to analysis	Associated network functions	Score^b^	Top canonical pathway	*P* value
Cell lysate proteins	Hereditary disorder, metabolic disease, molecular transport	48	Mitochondrial Dysfunction	3.65*E* − 04
Cell lysate and secretory proteins^a^	Lipid metabolism, molecular transport, small molecule Biochemistry	31	Gluconeogenesis I	4.67*E* − 04

^a^Earlier reported [7].

^b^A score of 2 or higher indicates at least a 99% confidence of not being generated by random chance and higher scores indicate a greater confidence.
